# Error Types of and Strategies on Learning Chinese Connectives: A Study on Chinese as a Second Language Learners’ Writing

**DOI:** 10.3389/fpsyg.2021.790710

**Published:** 2022-01-24

**Authors:** Lirui Zhang, Shaobo Sun, Shuangyun Yao

**Affiliations:** ^1^School of Foreign Languages, Xuchang University, Xuchang, China; ^2^Research Center for Language and Language Education, Central China Normal University, Wuhan, China

**Keywords:** Chinese connectives, Chinese as a second language, error types, learning strategy, corpus analysis

## Abstract

The correct use of connectives has great influence on language learners’ writing proficiency, while errors of connectives are common in foreign learners’ interlanguages. This study examines the types of errors that occur in native English-speaking learners’ Chinese writing, the possible causes for the errors, and the learners’ consequent learning strategies. The present research adopted corpora investigation, questionnaire survey, and focus-group interviews to examine the error types, causes of identified errors, and related learning strategies. Data analysis indicated that: (1) the main error types made by native English-speaking learners from high to low are misuse, overuse, mismatch, misplacement, and underuse of connectives; (2) causes related to intralingual transfer greatly contributes to the presence of errors; and (3) memory, social, and cognitive strategies were the most preferred, followed by metacognitive and compensation strategies, and then by effective strategies which were the least preferred. These findings showed that different strategies can be employed to cope with different errors in writing. The study further suggests that teachers and educators need to help native English-speaking learners find strategies that work best for them in terms of learning Chinese connectives.

## Introduction

Connectives are one-word items or fixed word combinations that express the relationship between clauses, sentences, or utterances in the discourse (Pander and Sanders, 2006, p. 33). Specifically, connectives are recognized as conjunctions (Halliday and Hasan, 1976), cohesive devices (Schiffrin, 1987), discourse markers (Fraser, 1999), and discourse units (Celle and Huart, 2007). They play an important role in language expression fluency as well as argumentation reliability in both spoken and written language (Hu and Li, 2015; Uccelli et al., 2015; Crossley et al., 2016) and in writing and reading (Ferstl and von Cramon, 2001; Crosson and Lesaux, 2013). Previous studies have reported positive correlations between the presence of connectives and writing quality. For example, Lee (2002) made a classroom inquiry about ESL students and found that the teaching of connectives could enhance students’ awareness of eligible writing. Mohammed (2015) argued that in the foreign language learners’ writing, it is not the presence or the absence of cohesive items that makes a text well-organized, rather it is the appropriate use of these conjunctive markers. Other studies have shown that connectives are especially challenging for language learners since even experienced writers have difficulties in using them correctly (Cheng and Tsang, 2021) and errors of connectives are common in foreign learners’ interlanguages. According to a study conducted by Gaskell and Cobb (2004), the error rate of using connectives is high in both English and Chinese writing produced by L2 learners. Lu (2000) also found that the error rate of function words, especially connectives, was above 60% in L2 Chinese writing. Hence, additional research on connective errors in non-native speakers’ writing is needed.

Chinese connectives refer to a collection of grammatical cohesive devices that are employed to convey the semantic relationships for the sake of discourse coherence (Lu, 2019). For instance, there are adversative relationships typically marked by connectives *danshi* “but,” the causal-effective relationship typically marked by *yinwei* “because” and *suoyi* “so,” the purposive relationship typically marked by *weile* “in order to,” etc. (The classification and grammatical patterns will be illustrated in Section “Classification and Grammatical Patterns of Chinese Connectives”). Chinese and English connectives share some commonalities, such as being present in large quantities, having complex semantic types, and high frequency of use, but differences with regards to how they are used still exist. Specifically, connectives are required in English, which is a hypotaxis-prominent language that emphasizes uniformity and integrity in sentence structure, whereas connectives are optional in some contexts in Chinese, which is a parataxis-prominent language that emphasizes the relevance of function and meaning (Lian, 2010, p. 73–84). Such differences make it difficult for native English-speaking learners to learn and use Chinese connectives. Given the lingua franca status of English in language education, research on the issue of learning and using connectives has mainly focused on the context of English education (Ma et al., 2017; Gong et al., 2018, 2020b,c). In particular, and in contrast to a large number of studies on connective acquisition in L2 English (e.g., Tapper, 2005; Lai, 2008; Mohammed, 2015; Tian et al., 2021), a paucity of pertinent studies has been conducted in the field of L2 Chinese acquisition. Among the few studies that focus on L2 Chinese connective acquisition, Ke (2005) made extensive research into L2 Chinese learners’ acquisitional patterns of 19 linguistic features and Zhang (2014) investigated the comprehension of 16 pairs of connectives by Chinese heritage and foreign language learners. However, previous studies mostly paid attention to acquisition patterns and error types but failed to explore the possible causes and learners’ learning strategies. At the same time, the majority concentrate on learners from Asian countries (e.g., Korea: Zheng, 2009; Thailand: Yang, 2016; Vietnam; Pan, 2013; Indonesia: Li, 2010). That is, there is a lack of comprehensive research on Chinese connective acquisition, especially in foreign learners from English-speaking countries.

In this research, we will investigate error types that occur in native English-speaking learners’ Chinese writing, examine possible causes for the errors, and inquire the learners’ relevant learning strategies. Furthermore, based on these points of examination, the study will put forward suggestions that will help teachers and educators better assist native English-speaking learners in finding out the strategies that work best for them in learning Chinese connectives.

## Literature Review

### Previous Studies on L2 Chinese Connective Acquisition

Many previous studies on L2 Chinese connective acquisition have studied the acquisitional patterns and error types, and most of the studies focused on single type of Chinese connectives of foreign learners from Asian countries. For instance, Zheng’s (2009) investigation of conditional connective errors made by Korean students revealed differences among errors in using conditional connectives. Pan (2013) investigated concessive connective errors made by Vietnamese learners. Zhang (2014) surveyed the comprehension of 16 pairs of connectives by Chinese heritage and foreign language learners and reported four categories of errors related to the usage of paired connectives: misplacement of connectives, mismatched pairs of connectives, absence of obligatory connectives, and order-reversed pairs of connectives. Yang (2013) examined the use of connectives by three Chinese as a second language learners in the United States and drew upon their written summaries on lesson contents presented in their textbooks.

The studies on L2 Chinese connective acquisition have mainly analyzed its typical error types, acquisition order, and acquisitional patterns of L2 Chinese learners. However, little attention has been paid to comprehensive investigation of typical errors in all kinds of connectives, the error cause, or learners’ learning strategies.

### Classification and Grammatical Patterns of Chinese Connectives

Chinese connectives are a type of conjunction. The function of which is to connect clauses and indicate logic-semantic relations between clauses and within complex sentences (Xing, 2016, p. 432). Given the important position of connectives in Chinese grammar, many different definitions and types of connectives have been presented by scholars (Xing, 2001; Huang and Liao, 2011; Yao, 2017). Among them, Xing’s (2001) classification, which includes coordinative, chronological, progressive, alternative, causal-effective, inferential, hypothetical, conditional, purposive, adversative, concessive, and negative-adversative connectives, is one of the most influential frameworks of Chinese connectives and is adopted in this research.

In this part, we will start with five basic grammatical patterns of Chinese connectives (1) Usage of a single connective, (2) Use of two collocated connectives, (3) Repetition of a single connective, (4) Use of two types of connectives in one semantic level, and (5) Use of two or more different pairs of connectives in multiple semantic levels. These five grammatical patterns are the basis for error analysis in the present study and are exemplified below:

(1) Usage of a single connective: In this pattern, the use of a single connective to indicate the logic-semantic relation of clauses in a complex sentence is common, as shown in Example 1 below.

Example 1:

我需要与爸爸好好交流一下，否则他会误解我。

Wo xuyao yu baba haohao jiaoliu yixia, fouze ta hui wujie wo.

I need with father well communicate one CL otherwise he will misunderstand me

“I need to talk with my father, otherwise he will misunderstand me.”

In this example, there is only one connective *fouze* “otherwise” which is used as a negative-adversative marker.

(2) Use of two collocated connectives: In this pattern, two connectives are typically used as a fixed collocation, as shown in Example 2 below.

Example 2:

周明虽然有仁慈的心肠，但是太偏颇了。

Zhou Ming suiran you renci de xinchang, danshi tai pianpo le.

Zhou Ming although have kind NOM heart but too biased PFV

“Although Zhou Ming is warm-hearted, he is too biased.”

In this example, the connectives *suiran* “although” and *danshi* “but” are juxtaposed in separate clauses, indicating the concessive-adversative relation between the clauses.

(3) Repetition of a single connective: in this pattern, one connective can be used repeatedly in several parallel clauses, as shown in Example 3 below.

Example 3:

无论他走多远, 无论他怎么逃避, 他的内心始终放不下。

Wulun ta zou duo yuan, wulun ta zenme taobi, ta de neixin shizhong fang bu xia.

No matter 3SG walk how far no matter 3SG how escape 3SG POSS heart always let NEG down

“No matter how far he goes and in what way he escapes, he cannot let it go.”

In the example above, *wulun* “no matter” is used twice in two clauses of one complex sentence, indicating a concessive relation.

(4) Use of two types of connectives in one semantic level: In this pattern, connectives with different semantic functions can be used together in one complex sentence to show two different logic relations of clauses at the same semantic level.

Example 4:

楼上是卧室, 平常即使没有人住, 可也都打扫的干干净净。

Loushang shi woshi, pingchang jishi meiyou ren zhu, ke ye dou dasao de ganganjingjing

Upstairs is bedroom usually even though no human live but also all clean NOM spotless

“Upstairs is a bedroom. It is swept clean everyday, even though nobody lives in.”

In the example above, the concessive connectives *jishi* “even though” and *ye* “also” are used with the adversative connective *ke* “but” within the same complex sentence, explicating two relations at the same time.

(5) Use of two or more different pairs of connectives in multiple semantic levels: in this pattern, two or more pairs of connectives with different semantic functions co-occur to show the logic-semantic relations among clauses on multiple semantic levels.

Example 5:

虽然这样做可以先打死一部分敌人, 但是如果敌人进行反击, 那够呛。

Suiran zheyang zuo keyi xian dasi yi bufen diren, danshi ruguo diren jinxing fanji, na gouqiang

although this way do can first kill some enemy but if enemy take fight back then terrible

“We can kill some enemies in this way at first, but it will be terrible if they fight back.”

In the example above, *suiran* “although” and *danshi* “but” are used in a pair, indicating the concessive-adversative relation of the first and the following two clauses, while *ruguo* “if” and *na* “then” are also used in a pair, indicating the hypothetical relation of the second and third clauses. This sentence consists of two pairs of connectives collocating with each other.

### Error Analysis Theory

Error analysis (EA) is a method used to investigate errors, including the causes of errors and the learning rules of foreign language learners, which makes foreign language teaching more effective and targeted (Corder, 1967). It is an important tool in language teaching pedagogy as it “helps teachers identify the sources of errors and take pedagogical precautions” (Khanom, 2014, p. 39). The most significant contribution of EA lies in its success in elevating the status of errors from undesirability to that of a guide to the inner working of the language learning process (Corder, 1974). Corder (1974) proposed that the EA procedures include collection, identification, description, explanation, and evaluation, and classified the errors in terms of the difference between the learners’ utterance and the reconstructed version into four categories: omission of some required elements; addition of some unnecessary or incorrect elements; selection of incorrect elements; and misordering of the elements. Further, Zhou (2007) described common grammatical errors in Chinese learning in detail, including addition, omission, misplacement, reference errors, and mixed errors.

Keshavas (1997) proposed that, the fields of EA can be divided into two branches, theoretical and applied. Theoretical EA is concerned with the process and strategies of language learning, which tries to investigate what is going on in the minds of language learners. While the applied branch is concerned with organizing remedial courses and devising appropriate materials and teaching strategies. It is worth noting that, the present study only concerns theoretical EA. To be precise, this study aims at investigating what is going on in the minds of language learners and what strategies language learners adopt in response to their errors.

### Learning Strategies

Language learning strategies are defined as “conscious mental and behavioral procedures that individuals engage in to gain control over their learning process” (Ortega, 2009, p. 208); “deliberate goal-directed attempts to manage and control efforts to learn the L2” (Oxford, 2011, p. 12); “procedures that facilitate a learning task” (Brown, 2006); and “activities consciously chosen by learners to regulate their language learning” (Griffiths, 2008, p. 87). Oxford (1990) sees the aim of language learning strategies as being oriented toward the development of communicative competence. Given the important role learning strategies play in foreign language learning, there have been several investigations into these strategies (Oxford, 2011; Rafik-Galea and Wong, 2011; Szyszka, 2017). Among them, the specific strategies adopted by students when learning L2 skills have been brought into focus (Oxford, 2011; Szyszka, 2017). Research has proved that appropriate learning strategies are important in helping students become more successful language learners, but still much remains to be investigated about what are applicable learning strategies that can be aimed at concrete language tasks. Oxford (1990) divides language learning strategies into two main classes, direct and indirect, which are further subdivided into six groups. Direct strategies include memory (strategies used for storage of information), cognitive (the mental strategies learners use to make sense of their learning), and compensation strategies (strategies that help learners overcome knowledge gaps and continue communication). Indirect strategies include metacognitive (strategies that help learners regulate their learning), affective (strategies concerned with the learner’s emotional requirements such as confidence) and social (strategies that lead to increased interaction with the target language) strategies.

Based on the EA procedures and through focus-group interviews, this study poses three research questions:

RQ1: What are the types of Chinese connective errors in native English-speaking learners’ writing?

RQ2: What are the possible causes for these errors?

RQ3: What strategic responses do English-speaking learners adopt in response to these connective errors?

## Methodology

### Participants

All participants of this study are Chinese as a second language (CSL) learners whose native language is English. To enhance the research generality, we selected the participants in line with the following criteria: (1) all participants are native English-speaking learners at intermediate and advanced levels, which can guarantee that they have Chinese connectives learning experiences; (2) they must study and live in mainland Chinese universities for at least 3 years so that they may employ all possible learning strategies; (3) there should be balance in the number of males and females to minimize the gender difference; and (4), the age of interviewees needs to be represented in the community of foreign learners. Therefore, a questionnaire survey was conducted amongst 86 native English-speaking learners to investigate the causes of errors. All these 86 native English-speaking learners were at the intermediate and advanced language level and from four mainland universities. To address the second research question, we selected and interviewed 10 of the 86 learners, four females and six males, to find out what strategies they use to address Chinese connective errors. According to Krueger (1994), the members in a focus-group interview should share similar characteristics so that they can feel comfortable with each other and engage in discussion. So, we chose 10 participants from the same university. Their ages range from 20 to 28 and all of them are at the intermediate and advanced language level. The participants have been assigned pseudonyms in order to protect their identity. Information such as gender, age, nationality, major, and language level can be seen in Table 1.

**TABLE 1:** Participants’ profiles.

	Name	Gender	Age	Nationality	Major	Language level
(1)	Jane	Female	23	United States	Chinese	Advanced
(2)	Frank	Male	23	United States	Chinese	Intermediate
(3)	Steve	Male	28	United Kingdom	Education	Advanced
(4)	Willy	Male	24	United States	History	Advanced
(5)	Anna	Female	21	Canada	Chinese	Intermediate
(6)	Marian	Female	20	Australia	Chinese	Intermediate
(7)	Sarah	Female	23	United States	Chinese	Advanced
(8)	Kenny	Male	20	United Kingdom	Chinese	Intermediate
(9)	Nelson	Male	28	Canada	News	Advanced
(10)	Louis	Male	23	United Kingdom	Education	Advanced

### Corpora

All the data are taken from two corpora: native English-speaking learners’ Chinese writing from the dynamic corpus of HSK composition and a self-compiled corpus constructed from the writing of 86 native English-speaking learners. The size of the HSK corpus is 300,321 Chinese characters and that of the self-compiled corpus is 203,247 Chinese characters, with the target connectives annotated.

HSK Dynamic Composition Corpus is a corpus of examination compositions written by intermediate and advanced Chinese language learners from different countries from 1992 to 2005. Its size can meet the requirements of the present study. The self-compiled corpus included the compositions written by native English-speaking learners from four mainland universities in the past 3 years.

### Data Collection

In this study, the error analysis was conducted in accordance with the procedure proposed by Corder (1974). First, Chinese written works composed by native English speakers from the above-mentioned corpora were collected. The collection process involved determining the learners’ native language backgrounds which was done by checking the language background of the participants to make sure that all of them were native English speakers. Second, we analyzed the texts with a focus on identifying all connectives used, and consequently identified 1321 sentences with connective errors. Third, we described the error types by comparing the incorrect usage of the Chinese connectives with the correct usage. This involved classifying the errors and assigning a grammatical description of each error. Then, the errors were explained by attempting to identify the causes of the errors. Finally, the errors were evaluated in detail by assessing them with a focus on finding out the participants’ learning strategies.

A questionnaire survey was conducted among 86 native English-speaking learners to get a full picture of what the possible causes for the errors were. The questionnaire was designed by referring to Richards’ (1974) identification of various strategies associated with developmental errors, which includes (1) overgeneralization, a device used when the items do not carry any obvious contrast for the learner; (2) ignorance of rule restrictions occurs when rules are used in context where in target language usage they do not apply; (3) incomplete application of rules involves a failure to learn the more complex types of structure; (4) false concepts hypothesized refers to errors derived from faulty understanding of target language distinctions. Apart from the given options, we also allowed space for the participants to add their possible causes (see Appendix 1). According to scale development standard proposed by Shi et al. (2012), 10 experts were invited to assess the content validity from tow perspectives of item-level content validity index (I-CVI) and scale-level content validity index based on the average (S-CVI/Ave). The results showed that I-CVI > 0.8 and S-CVI/Ave = 0.94, the content validity was satisfying. All the data were collected and analyzed before the focus-group interviews.

After collecting and analyzing the error causes from the questionnaire survey, we conducted focus-group interviews with 10 of the 86 native English-speaking learners in an informal group discussion. A focus-group interview is reliable for “exploring what individuals believe or feel as well as why they behave in the way they do” (Rabiee, 2004, p. 655). It was used because the naturalistic conversational situation it creates helps to obtain authentic and rich data (Abednia et al., 2013; Gong et al., 2020a), and its focus on “ideas and feelings that individuals have about certain issues” (Rabiee, 2004, p. 656) was in line with our research objectives. The focus-group interview lasted for 4 h. During the interview, four interviewers had a free but purposive discussion with the participants to elicit the research issues, then all questions were discussed by all participants. We assume that the result is credible because “the type and range of data generated through the social interaction of the group are often deeper and richer than those obtained from one-on-one interviews” (Thomas et al., 1995, p. 207) and “focus groups could provide information about a range of ideas and feelings that individuals have about certain issues, as well as illuminate the differences in perspective between groups of individuals” (Rabiee, 2004, p. 656). The interview questions mainly included: (1) Which of the five types of connective errors are you most likely to make? (2) What is your biggest challenge in learning Chinese connectives? (3) What strategic responses do you adopt in response to these connective errors? And (4) What strategies have worked for you? The interviewers were proficient in both Chinese and English. When the participants had difficulties expressing themselves in Chinese, they were allowed to speak English, which ensures that they could fully express their views. All the interviews were audio recorded, and transcribed verbatim in Chinese by interviewers (not including the authors). After the interviewers completed the transcription, the participants were asked to review and verify the transcriptions in order to guarantee accuracy of the transcribed data.

## Results

After checking all 1,321 sentences with connective errors, five typical error types were identified: (1) mismatch of connectives, (2) misuse of connectives, (3) underuse of connectives, (4) overuse of connectives, and (5) misplacement of connectives. The results of the 86 native English-speaking learners’ questionnaires revealed the most likely causes for the typical error types and the focus-group interview manifested what learning strategies were preferred by the participants.

### Typical Errors in Connective Usage

Following the error types classified by Corder (1974) and Zhou (2007), and in combination with the data from our results, five types of errors were identified. All examples in this paper are from the above-mentioned HSK dynamic composition corpus and the self-compiled corpus unless otherwise specified. The five typical errors are exemplified as follows:

(1) E1: mismatch of connectives – refers to the use of connectives in pairs that do not match.

Example 1:

他不仅要保护自己, 而是要保护所有人。

*Ta bujin yao baohu ziji, ershi yao baohu suoyou ren

He not only protect himself but protect everyone.

He had to protect not only himself but everyone.

In this sentence, the progressive connective *bujin* (not only) cannot be used with the adversative connective *ershi* (but) in a pair; *ershi* (but) should be replaced with *erqie*(also) which would make a complete pair.

(2) E2: misuse of connectives – refers to the use of inappropriate connectives to indicate the logic-semantic relation of a complex sentence.

Example 2:

他受伤了, 于是明天可能不会来。

*Ta shoushang le, yushi mingtian keneng buhui lai

He hurt so he tomorrow won’t come.

He is hurt and may not come tomorrow.

This is a typical causal-effective complex sentence. Both *yushi* and *suoyi* are effective markers, but *yushi* can only be used for past events, not for a future event (Zhao, 2003), which makes it inappropriate. The second clause “he may not come” is a future event, so *yushi* should be replaced by the effective marker *suoyi* or *yinci*.

(3) E3: underuse of connectives – refers to the omission of connectives in a place where they should be used.

Example 3:

他不但外表出众，人品很好。

*Ta budan waibiao chuzhong, renpin hen hao

He not only appearance outstanding character good.

He is not only outstanding in appearance, but also good in character.

In Chinese, *budan* (not only) is commonly matched with *erqie/ye*/*hai* (but also/even) to form a fixed collocation (Lü, 2005, p. 94). In such patterns, *budan* is optional but the latter is not.

(4) E4: overuse of connectives – this error is caused by using connectives where they should not be used.

Example 4:

重要的原因是因为这些孩子是在幼儿园学的。

*Zhongyao de yuanyin shi yinwei zhexie haizi shi zai youeryuan xue de

Important reason is because these children in kindergarten learn.

The important reason is that the education of these children was received in kindergarten.

In this sentence, the semantic function of *zhongyaodeyuanyin* (the important reason) and that of *yinwei* (because) overlap. This means that *yinwei* should be omitted.

(5) E5: misplacement of connectives – refers to the use of the connectives in improper positions in a complex sentence.

Example 5:

王明不但取得了好成绩，而且他的队友都取得了好成绩。

*Wang Ming budan qude le hao chengji, erqie ta de duiyou dou qude le hao chengji

Wang Ming not only get good results but also his teammates all get good results.

Not only did Wang Ming get good results, but all of his teammates also got good results.

In this example, “Wang Ming” is the subject of the first clause but not the whole sentence, so the progressive connective *budan* should be placed before the first clause (Lü, 2005, p. 94).

**FIGURE 1:**
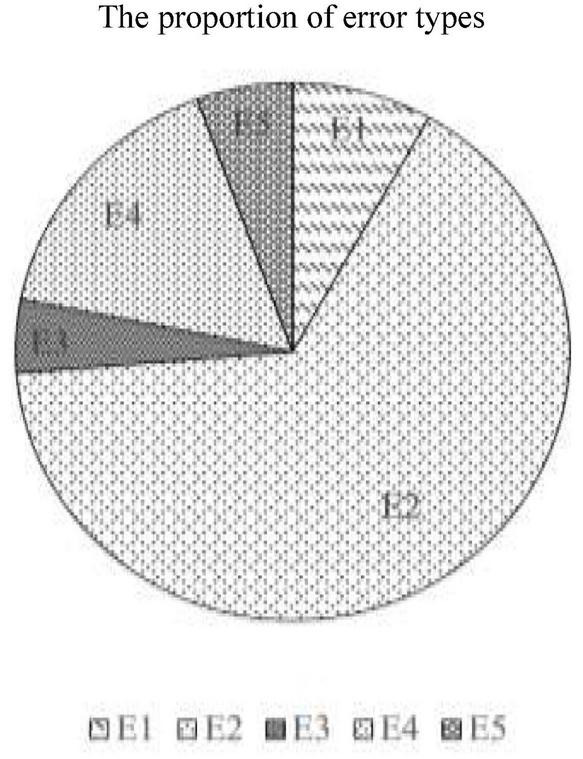
The proportion of error types. E1, mismatch of connectives; E2, misuse of connectives; E3, underuse of connectives; E4, overuse of connectives; E5, misplacement of connectives.

The proportion of each error type is shown below in Figure 1.

As seen in Figure 1, the proportion of E2 is highest at more than 65.6%. E4 is in second place at 16.3%. E1 and E5 are at the third and fourth place with the rate of 8.2 and 5.6%, respectively, and E3 is the least at only 4.4%.

### Causes of Typical Errors

In view of the identified errors, we created a questionnaire survey that was administered to 86 native English-speaking learners to find out the likely causes behind these connective errors. This is the fourth step of EA previously outlined in Section “Data Collection” which sets the foundation for the following focus-group interviews. The results indicate the causes in two domains raised by Erdogan (2005), namely interlingual transfer and intralingual transfer. Khanom (2014) listed the specific causes in these two domains as (1) L1 interference in interlingual transfer, (2) overgeneralization, (3) ignorance of rule restrictions, (4) incomplete application of rules, and (5) false concepts hypothesized in intralingual transfer. These causes and domains are outlined in Table 2 below. The first column lists the error code, with the first error cause marked as C1, the next as C2, and so on. The second column contains error causes listed in the questionnaire, the third column lists the domains of error causes, and the last column lists the proportion of each error cause.

**TABLE 2:** Causes of typical errors.

Code	Error causes in the questionnaire	Domains	Proportion (%)
C1	The meanings of some connectives are too close to distinguish their differences.	Intralingual transfer	55.9
C2	Lack of practice in using some connectives.	Intralingual transfer	53.5
C3	I apply the wrong Chinese grammar rules.	Intralingual transfer	48.8
C4	Trying to avoid using such complex connectives for fear of making mistakes.	Intralingual transfer	46.5
C5	I don’t understand the whole sentence.	Intralingual transfer	44.1
C6	There is no equivalent usage in the mother tongue.	Interlingual transfer	41.7
C7	Drawing a false equivalence between an English connective and a Chinese one that shares a similar meaning.	Interlingual transfer	41.7
C8	The wrong judgment was made by applying the grammar rules of the mother tongue.	Interlingual transfer	29.1
C9	Different ways of thinking result in different views on the relationship between clauses.	Interlingual transfer	25.2
C10	The wrong usage is often used by native Chinese speakers.	Intralingual transfer	21.3
C11	I am not willing to learn some connectives because they are useless.	Intralingual transfer	17.3
C12	Such usage has appeared in a certain book.	Intralingual transfer	9.4
C13	The teacher’s explanation of such connectives is not clear enough to be understood.	Intralingual transfer	9.4
C14	Such usage has been seen in films, television works, and online media.	Intralingual transfer	7.9

Compared with the findings of Cao (2013) and Yang (2013), who claimed that L1 transfer was the primary driving force of connective errors, it is noteworthy that intralingual transfer plays a big part in the error causes in this study. Therefore, we emphasized the strategies adopted by learners to address intralingual errors and investigated these applicable strategies that were used to reduce intralingual errors.

### Strategic Responses to Connective Errors

The findings of the focus-group interview showed that the participants adopted memory, cognitive, social, metacognitive, compensation, and affective learning strategies in response to their connective errors. As “the results of focus-group interviews can be presented in uncomplicated ways using lay terminology supported by quotations from the participants” (Rabiee, 2004, p. 656), we will illustrate how the participates employ learning strategies in response to connective errors in the following sections by using quotes from their interviews.

#### Direct Strategies

Language learning strategies that are directly involved in language learning are called direct strategies. Oxford (1990, P. 17) identified memory, cognitive, and compensation strategies as direct strategies.

##### Memory Strategies

Memory strategies help students store and retrieve new information; cognitive strategies help them understand and produce new language by many different means, and compensation strategies allow them to use the language despite gaps in knowledge. The most commonly used strategy by the 10 participants was the memory strategy, which is thought to be the most direct and effective strategy for the learning of connectives. Based on the analysis of the interview results, we found that all interviewees (10/10) adopted methods like memorizing typical example sentences, associative memory, and network media assisted memory to deal with errors such as the mismatching of connectives (E1), selection of incorrect connectives (E2), and omission of connectives (E3). Jane, who often mismatched connectives in the early stages of learning, did so due to being unfamiliar with fixed collocations. In the Excerpt (1) below, she shares her experience:

[1]

Well, at first, I was not really familiar with Chinese connectives and the only method I adopted was trying to use and memorize them again and again. Fortunately, our teachers usually analyze the sentences in our books and teach us more typical sentences, so I would try to recite these sentences. And, you know, in this way, I try to understand and memorize the connectives in them. Then, gradually, I memorized some fixed collocations and came to know how to use these connectives.

Willy was another participant who also adopted the method of memory strategy. However, based on the existing connectives in the sentences, he would associate related or similar connectives and memorize them together. In Willy’s view, this associative memory method helped him memorize connectives faster and more firmly. A few students (3/10) thought that rote learning was very boring and inefficient, so they turned to the method of network media associated memory. Louis is one of them. Louis shared that he sometimes proactively searched for some example sentences with certain connectives on Chinese websites like Baidu and copied the typical sentences for reading and reciting.

##### Cognitive Strategies

Eight students adopted the cognitive strategy in this interview. The cognitive strategy refers to the “manipulation or transformation of the target language by the learner” (Oxford, 1990, p. 43) or general mental processing (Schmitt, 2000). Specifically, cognitive strategies include various methods such as strengthening practice, inferential reasoning analysis regarding the meaning of connectives and sentences, and paradigm shifting, which mainly help foreign students overcome errors of misuse (E2), overuse (E4), and misplacement (E5). Some students (8/10) thought that practice was a useful method, which can be summarized as “practice makes perfect.” For example, Frank mentioned that:

[2]

I prefer doing exercises to learn Chinese grammar. You know, I always do a lot of grammar practices after class, including the exercises given by our teachers, and other sentence-making practices I find from other reference books. For example, I learned a connective in our class today, and I will use it to make sentences after class. Then, I’ll ask my teachers or my Chinese friends whether they’re correct. Well, I think this is a good way for me to learn how to use Chinese connectives.

The description of connective error types mentioned in Figure 1 indicates that the misuse of connectives largely existed in foreign students’ writing (accounting for 65.6%). From the causes in Table 2 (see Section “Causes of Typical Errors” above), we can infer that the main reason for connective errors is that learners could not distinguish the meaning and usage of similar connectives, nor could they understand the logical relationship between clauses. For instance, once in a test, Steve misused *suoyi* (‘so’) as *yushi* (‘then’) (see e.g., 2 in Section “Typical Errors in Connective Usage”), and he felt very confused about the distinction between the two connectives. Later, with the teacher’s help, he carefully analyzed the complex sentences containing these two connectives and came to understand the logical relationship between the clauses. In the end, he gained insight into the differences between them. In addition, given the impact of the negative transfer of the native language (English), Jane believed that a shift in thinking, that is, learning Chinese sentences in a Chinese way is essential.

##### Compensation Strategies

Some interviewees (3/10) mentioned compensation strategies during their interviews. Compensation strategies belong to the group of direct strategies and involve using the new language for comprehension or production (Oxford, 1990) and are intended to “help learners with limited or complete absence of vocabulary knowledge and grammar” (Letchumanan et al., 2016, p. 175). The interview results indicated that the three students hope to deal with errors such as mismatch (E1), misuse (E2), and overuse (E4) by using the dictionary and guessing the meaning of connectives. Nelson is one of these students who, when faced with two similar connectives, finds it hard to make a choice and then looks up the connectives in the dictionary. He expressed that consulting dictionaries can help him deal with some errors. The following is his opinion:

[3]

You know, er… the difference between some connectives you know, are…are very small. Even with the dictionary, sometimes, I still can’t distinguish them. Well, this really er… this makes me feel confused.

In addition, a student named Anna also mentioned the method of guessing. When feeling confused about which connectives to choose, she would first judge which categories of connectives were needed, then guess the specific ones. Although compensation strategies were also adopted by these students to address connective errors, this class of strategies was not their initially preferred choice amongst all the available strategies.

Results from the interview show that, in terms of direct strategies, the ten participants tended to adopt the memory strategy followed by the cognitive strategy, and then the compensation strategy, despite the effect of the compensation strategy being limited.

#### Indirect Strategies

According to Oxford (1990, p. 135), indirect strategies consist of metacognitive, affective, and social strategies. All these strategies are indirect because they support language learning without involving the target language directly.

##### Social Strategies

Social strategy, which involves interaction with other people to improve vocabulary learning (Oxford, 1990; Schmitt, 2000), is one of the most frequently used strategies. Nine out of ten participants said they have adopted this strategy. Through exchanges with their classmates and Chinese friends, the participants obtained a communicative and interactive environment for learning and using Chinese connectives. Marian and Anna were classmates and good friends. When learning Chinese, Marian encountered serious challenges in using some connectives. She often discussed her problems with Anna after class and then practiced the usage of some connectives, sometimes even communicating in Chinese. In the excerpt (4) below, Marian shares her experience:

[4]

For me, the most difficult things in Chinese learning are the connectives and complex sentences. I was once very upset. Fortunately, my friend Anna always encouraged me. By discussing and practicing with her, I made good progress in learning Chinese. She really helped me a lot, and she is my good friend as well as my good teacher.

During the interview, five students said that they hoped to make friends with Chinese people because communicating with native speakers could provide them with real-life Chinese context. Kenny, for example, said that even though he could understand the usage of connectives taught in class, he could hardly use them properly in daily conversation. Therefore, he and his Chinese friends met twice a week to chat or dine together, during which he endeavored to communicate in Chinese and also paid special attention to how his friends used connectives.

##### Metacognitive Strategies

Another indirect strategy is the metacognitive strategy. Metacognitive strategy includes the methods used to oversee, regulate or self-direct language learning (Hismanoglu, 2000). Five participants made it clear that they had used this strategy. In the process of learning connectives, these students do not only make study plans for themselves but also evaluate their learning results by sorting out the wrong sentences they had made so as to improve their study plans in real time. Frank studied very hard and made a detailed plan for himself in order to master Chinese connectives better. In the interview, he showed us a list of study plans which included detailed steps such as studies, practices, reviews, and applications. In the excerpt (5) below, Sarah shares that she has the habit of sorting out wrong sentences, holding the view that by analyzing the incorrect sentences she had made, she could not only find her weak points but also evaluate her own learning results. This allows her to adjust her learning focuses and strategies in real time.

[5]

Because I often use connectives incorrectly, I have made a plan for myself to review connectives. And also, … I will extract the mistakes related to connectives in previous exercises to see which kinds of connectives I am more likely to misuse. So, … in the future, I will practice these connectives more.

##### Affective Strategies

In addition to social strategies and metacognitive strategies, four international students also mentioned affective strategies. Affective strategies are concerned with the learner’s emotional requirements. For example, as Marian mentioned above in the excerpt (4), she felt depressed when she met some setbacks in her study. With the encouragement of her good friend Anna, Marian regained her confidence, gained the motivation to study, and gradually overcame some difficulties in learning connectives.

From these interviews, it can be concluded that among indirect strategies, students prefer social strategies, followed by metacognitive strategies and affective strategies.

## Discussion

This study investigated the types of errors that native English-speaking learners make when using Chinese connectives, the possible causes, and the strategic responses they adopted in response to the errors. Most previous studies published in English have investigated the use of connectives in ESL and EFL learners’ writing (Tapper, 2005; Lai, 2008; Mohammed, 2015; Tian et al., 2021), the results of which have shown that the underuse, overuse, and misuse of English connectives are common. In view of the peculiarity of Chinese connectives, and based on the corpus investigation, in addition to the above-mentioned misuse (E2), underuse (E3), and overuse (E4) of connectives, we identified two more error types: mismatch (E1) and misplacement (E5) of Chinese connectives. Despite the low proportion (see Figure 1 in Section “Typical Errors in Connective Usage”), E1 and E5 still deserve our special attention because “one of the unique features of Chinese connectives is that they often seem to occur in pairs and in an orderly fashion as correlatives” (Lu, 2019, p. 559) and the distribution of Chinese connectives may be a function of their syntactic position in a sentence (Cao, 2013). Such uniqueness does not exist in English and this makes these two types of errors unavoidable in native English-speaking learners’ writing. Thus, it is necessary for teachers or educators to find appropriate learning strategies to help native English-speaking learners overcome these two special error types. According to our survey, the proportion of each error type from high to low is: E2, E4, E1, E5, and E3. The proportion of E2 is highest at more than 65.6%, which reveals that selecting correct Chinese connectives while writing is the biggest challenge for native English-speaking learners. This result is consistent with the conclusion of Altunay (2009) who found that misuses of some connectives were common. E4 is in the second most common error accounting for 16.3%, showing that native English-speakers tend to add connectives in sentences where they should not be used. The overuse of connectives was also found in other studies. For instance, Tapper (2005) found that advanced Swedish EFL learners tended to overuse adverbial connectives when compared to American university students. Lai (2008) also suggested that unskilled learners used connectives more frequently than the skilled ones through investigating the use of discourse connectors in the writing of EFL undergraduate writers from Chinese Taiwan. Although the result of this study is largely consistent with previous studies on English connective acquisition, what we attempt to stress in this study is having a thorough understanding of what strategies are adopted by native English-speaking learners and helping them to find the corresponding strategies best suited for learning Chinese as a second language, especially in terms of connectives (Gong et al., 2021).

After identifying and describing the errors, we explored the possible causes behind them through a questionnaire survey before investigating the learning strategies. The results offered fresh insights into Chinese connective learning. Negative cross-linguistic influence has been unanimously recognized by researchers as an important cause of errors (Lu, 2019); however, this study shows that intralingual transfer plays a big part in the cause of errors. This gives a hint that in the teaching of Chinese connectives, we should no longer overstress the L1 transfer as we used to but put certain emphasis on intralingual transfer.

As this study concerns theoretical EA, rather than simply listing the typical error types and causes, we went further to identify what learning strategies are employed by the participants in response to connective errors. In direct strategies, memory strategies were directed at error cause of C1 and C3 (see Table 2 in Section “Causes of Typical Errors”) and adopted by all participants (10/10) in response to error types E1, E2, and E3. Cognitive strategies could be used to solve the problems caused by C2. Since cognitive strategies “involve the element of practice, and practice promotes internalization of vocabulary items” (Letchumanan et al., 2016) and were applied by participants (8/10) to cope with all five types of errors, especially E2, E4, and E5. From some participants’ (3/10) viewpoints, compensation strategies could be risky and not effective, but consulting dictionaries helped them understand the meanings and usages of connectives, thereby reducing errors caused by C1, C6, C12, and C13. This is similar to what has been reported by Schmitt (1997), who conducted a study on the multiple uses of vocabulary learning strategies and concluded that, “the use of bilingual dictionary was the most used strategy among Japanese students and this was followed by guessing meaning in context.” Indirect strategies are useful in virtually all language learning situations and are applicable to all language skills (Oxford, 1990, p. 135). The most common indirect strategies employed by participants (9/10) are social strategies, which relate to causes of C10 and C14 and are effective in generally reducing connective errors. Half of the participants (5/10) showed their tendency to use metacognitive strategies. As one of the indirect strategies, metacognitive strategies cannot be used to reduce one or more concrete kinds of errors but can help language learners improve their connective acquisition overall. As Hismanoglu (2000, p. 35) notes, “all language learners use language learning strategies either consciously or unconsciously when processing new information and performing tasks.” Affective strategies, which could be useful to reduce the errors caused by C4 and C11, were unconsciously adopted by the participants (4/10). The findings reveal that participants may adopt one or more strategies in response to the same type of error, and one strategy may be employed in response to one or more types of errors. This falls in line with the findings in Rafik-Galea and Wong (2011), which reveals that the learners used multiple strategies to learn vocabulary. In addition, Rafik-Galea and Wong (2011) surveyed adult foreign language learners’ preferences for vocabulary learning strategies. They found that the five categories of vocabulary learning were cognitive, compensation, metacognitive, memory, and social categories. Among them, cognitive strategies were highly preferred by the learners and metacognitive were the least preferred. The reason why this result is different from ours is that we are focusing on connective learning strategies and not the overall vocabulary.

In terms of the findings, this study further suggests that teachers and educators need to assist learners in taking advantage of their preferred strategies to cope with the targeted errors. For instance, the findings show that intralingual transfer plays a big part in error causes and that the top two strategies are memory and social strategies. The results suggest that we need to adopt proper methods of memory strategies to solve errors caused by intralingual transfer. For example, the method of lexical chunk teaching, which emphasizes recitation of phrases, fixed phrases and other lexical chunks, can be introduced in connective teaching. Furthermore, the lexical chunks themselves are prefabricated and the whole block can be extracted so that learners can directly extract lexical chunks of relevant connectives in their writing (Zhang, 2017). This is useful in reducing connective errors, especially E1, E2, and E3 which are caused by intralingual transfer. In addition, when teaching connectives, teachers traditionally emphasize the meanings and different usages of connectives. However, the findings of this study revealed that using connectives in daily life is an effective way to improve Chinese connective learning. Thus, contextualization is required to help learners distinguish the pragmatic functions of Chinese connectives. We suggest that teachers adopt social strategies, such as assigning some tasks for learners to accomplish in collaboration with native speakers, to encourage them to communicate more with Chinese people and create conditions in which learners can learn and practice connectives by exposing themselves to the native language environment. Learning connectives from native speakers in the real world is undoubtedly an efficient way to decrease connective errors, especially the unique E1 and E5 errors in Chinese connective acquisition.

## Conclusion

The purpose of the current study was to investigate the Chinese connective error types in native English-speaking learners’ writing and the strategic responses they adopted in response to these connective errors. The results of this investigation showed that the main error types made by native English-speaking learners from high to low were misuse, overuse, mismatch, misplacement, and underuse of connectives. This study also found that the causes related to intralingual transfer greatly contributed to the presence of errors, and the participants employed both direct and indirect strategies to cope with connective errors. The direct strategies had a higher degree of pertinence and could be used to reduce some concrete types of errors caused by intralingual transfer while indirect strategies were more suitable for improving overall connective acquisition. The most preferred strategies employed by English-speaking learners were memory, social, and cognitive strategies, followed by metacognitive and compensation strategies, and affective strategies were the least preferred ones. These findings suggested that different strategies can be employed to cope with different errors in writing, and teachers and educators need to help native English-speaking learners find the strategies that work best for them in learning Chinese connectives.

A limitation of this study is that the investigation was only conducted with native English-speaking learners, and any generalizations of the findings to all CFL and CSL learners should be undertaken with caution. This study was based on corpus analysis and focus-group interviews. The results would be helpful to provide some general information about the Chinese connective acquisition of foreign learners. Although the data were collected by rigorously abiding by the error analysis procedure (Corder, 1974) and focus-group interview analysis (Rabiee, 2004), which are widely-accepted procedures in the academic field to enhance the trustworthiness of the research results, what was reported might be different from what was enacted in the actual context. Beyond that, we used in-depth group interviews in which participants are selected because they are purposive, but they are not necessarily representative and thus results may not be generalized to all foreign learners.

The main contribution of this study is that the results reveal typical Chinese connective errors, their possible causes, the learning strategies adopted by native English-speaking learners in response and their internal connections, which provide insights for teachers and educators to make better teaching procedures, and therefore has significant theoretical and practical value. Further studies can be conducted to explore grammatical errors in foreign learners writing with different native language backgrounds and explore the learners’ learning strategies from different perspectives (Tsung and Gong, 2021).

## Data Availability Statement

The original contributions presented in the study are included in the article/supplementary material, further inquiries can be directed to the corresponding author.

## Ethics Statement

Written informed consent was obtained from the individual(s) for the publication of any potentially identifiable images or data included in this manuscript.

## Author Contributions

LZ was responsible for providing the overall idea. SS was responsible for data analysis. SY was responsible for experimental design. All authors contributed to the article and approved the submitted version.

## Conflict of Interest

The authors declare that the research was conducted in the absence of any commercial or financial relationships that could be construed as a potential conflict of interest.

## Publisher’s Note

All claims expressed in this article are solely those of the authors and do not necessarily represent those of their affiliated organizations, or those of the publisher, the editors and the reviewers. Any product that may be evaluated in this article, or claim that may be made by its manufacturer, is not guaranteed or endorsed by the publisher.

## Appendix 1: Questionnaire

### Questionnaire for Error Causes

#### Part 1

Gender:
Age:
Nationality:
Length of Chinese learning:

#### Part 2

Tick your error causes

() (1) There is no equivalent usage in the mother tongue;
() (2) Drawing a false equivalence between an English connective and a Chinese one that share the similar meaning;
() (3) Wrong judgment made by applying the grammar rules of the mother tongue;
() (4) The meanings of a group of Chinese connectives are too close to distinguish their differences;
() (5) Apart from the connectives, the whole sentence is difficult to understand;
() (6) The wrong usage has appeared in a certain book;
() (7) The teachers’ explanation of these connectives was not clear enough to be understood;
() (8) Different ways of thinking result in different views about the relationship between the Chinese clauses;
() (9) Lack of practice;
() (10) Trying to avoid using connectives with complex meanings for fear of making mistakes.

#### Part 3

Please write down other possible causes:
